# PuraStat for gastrointestinal bleeding management: Effective approach for endoscopic prevention and rescue therapy

**DOI:** 10.1055/a-2550-6600

**Published:** 2025-04-04

**Authors:** Elettra Merola, Giovanni Mario Pes, Maria Pina Dore

**Affiliations:** 1 9312 Dipartimento di Medicina, Chirurgia e Farmacia, University of Sassari, Sassari, Italy



We read with great interest the study by Maselli et al. 1 about the potential role of PuraStat in endoscopic management and prevention of gastrointestinal bleeding. This real-world investigation, based on an Italian registry of 401 patients treated with this novel hemostatic agent, provides valuable insights. The relatively large cohort and the prospective design convincingly demonstrate the efficacy and safety of PuraStat for managing iatrogenic bleeding and preventing delayed post-procedure hemorrhage.

We fully agree with the authors about the utility of PuraStat for both active “oozing” bleeding and prevention of delayed rebleeding, particularly following challenging polypectomies. In our clinical practice, we often prefer powder-based hemostatic agents for diffuse bleeding; however, their utility is frequently hindered by risk of clogging and impaired endoscopic visibility.

Beyond these observations, we would like to highlight a critical aspect of the study by Maselli et al. 1 about application of PuraStat in cases of non-iatrogenic active gastrointestinal hemorrhage. Existing data on this indication in gastrointestinal endoscopy are scarce, with limited reports such as its use alongside ligation for active diverticular bleeding in the colon 2 . A key innovation of the study by Maselli et al. 1 lies in the use of PuraStat for active “oozing” bleeding as well as for severe acute hemorrhage as a secondary treatment. However, detailed descriptions of the five cases of “arterial spurt” bleeding included in the analysis were not provided in the manuscript, and lack of this information limits reader ability to clearly understand what characteristics of the active gastrointestinal bleeding lesions could really benefit from PuraStat treatment. Because the authors declare that in these cases PuraStat was used in conjunction with other hemostatic techniques (mechanical, injective, or thermal), it can be inferred that the approach was aimed at preventing delayed rebleeding, resulting in a technical success (no rebleeding reported in **Table 4** ).

In conclusion, we concur with the authors about the promising and expanding role of PuraStat in management of gastrointestinal bleeding. Nonetheless, only prospective comparative studies can definitely establish its indications, efficacy in bleeding control and prevention, and impact on major clinical outcomes such as hospitalization length, morbidity, and mortality rates.

**Publication note**

Letters to the editor do not necessarily represent the opinion of the editor or publisher. The editor and publisher reserve the right to not publish letters to the editor, or to publish them abbreviated or in extracts.
